# L2-CalSat: A Calibration Satellite for Ultra-Sensitive CMB Polarization Space Missions

**DOI:** 10.3390/s21103361

**Published:** 2021-05-12

**Authors:** Francisco J. Casas, Enrique Martínez-González, Juan Bermejo-Ballesteros, Sergio García, Javier Cubas, Patricio Vielva, Rita B. Barreiro, Angel Sanz

**Affiliations:** 1 Instituto de Física de Cantabria (IFCA), CSIC-UC, Avda. de los Castros s/n, 39005 Santander, Spain; martinez@ifca.unican.es (E.M.-G.); vielva@ifca.unican.es (P.V.); barreiro@ifca.unican.es (R.B.B.); 2 Instituto Universitario *Ignacio Da Riva* (IDR/UPM), Universidad Politécnica de Madrid, Plaza Cardenal Cisneros, 3, 28040 Madrid, Spain; juan.bermejo@upm.es (J.B.-B.); sergio.garcia@upm.es (S.G.); j.cubas@upm.es (J.C.); angel.sanz.andres@upm.es (A.S.)

**Keywords:** CMB polarization, formation flight, CubeSat, calibration, B-modes

## Abstract

In this work, the use of a calibration satellite (L2-CalSat) flying in formation with a Cosmic Microwave Background (CMB) polarization mission in an orbit located at the second Lagrange point, is proposed. The new generation of CMB telescopes are expected to reach unprecedented levels of sensitivity to allow a very precise measurement of the B-mode of polarization, the curl-like polarization component expected from gravitational waves coming from Starobinski inflationary models. Due to the CMB polarized signal weakness, the instruments must be subjected to very precise calibration processes before and after launching. Celestial sources are often used as external references for calibration after launch, but these sources are not perfectly characterized. As a baseline option, L2-CalSat is based on the CubeSat standard and serves as a perfectly known source of a reference signal to reduce polarization measurements uncertainty. A preliminary design of L2-CalSat is described and, according to the scanning strategy followed by the telescope, the influence of the relative position between the spacecrafts in the calibration process is studied. This new calibration element will have a huge impact on the performance of CMB space missions, providing a significant improvement in the measurements accuracy without requiring new and costly technological developments.

## 1. Introduction

The new generation of Cosmic Microwave Background (CMB) telescopes have reached unprecedented levels of sensitivity allowing the estimation of the cosmological parameters, in many cases, with sub-percent precision. However, there is a unique piece of information present in the CMB polarization B-mode, the curl-like component expected from the imprint left by the primordial gravitational waves generated during the inflationary period of the universe, that remains undetected. Up to date the only information we have on this signal, parametrized by the tensor-to-scalar ratio *r*, is that it is very weak with the best upper limit given by r<0.044 at the 95% CL [1] (see also [2,3]). Its detection represents a significant challenge from the instrumental and data analysis point of views (see  [4] for a review). From the point of view of fundamental physics, it has profound implications on the standard model of cosmology, in particular to confirm the inflationary episode in the very early universe [5], and furthermore as a fundamental test of Eintein’s General Relativity theory (see e.g., [6,7,8,9]).

The amplitude of this signal is at least three orders of magnitude fainter than that of the CMB temperature. Instruments on-board satellites are in excellent environmental conditions to measure the B-mode signal over the full sky. However, due to the signal weakness, the instruments must be subject to precise calibration processes before and after launching. Additionally, the scanning strategy chosen to cover the full sky plays an important role in measuring repeatedly the same sky area in different times and with different orientations of the focal plane, helping to mitigate systematic errors induced by the instruments.

**Figure 1:**
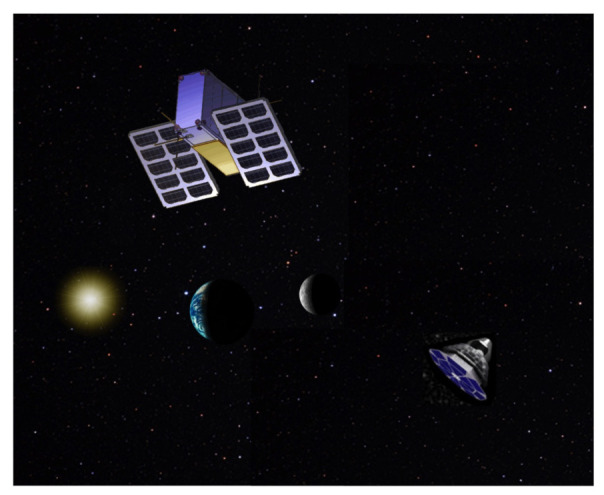
Artist’s view of two satellites in formation flying. Nearby to the left, L2-CalSat, the proposed calibration system hosted by a 6U CubeSat, while to the right and in the distance a space satellite for CMB polarization measurements is visible.

Celestial sources are often used as an external reference for calibration after launch, but the characterization of these sources is not sufficiently good to match the calibration requirements of CMB polarization experiments (see e.g., [10,11] for Tau A polarization angle uncertainties). In this paper we study the concept of a novel calibration system for the next generation of CMB polarization space missions, based on the use of a calibration satellite (L2-CalSat) flying in formation with a CMB telescope (CMB-Sat), in an orbit located at the second Lagrange point (Figure 1). During the calibration periods, L2-CalSat keeps the calibration sources within view of CMB-Sat, emitting purely polarized microwave radiation from the telescope’s far-field. As a result, the observations will benefit, among other things, from the absolute calibration of the polarization angle and the detectors beam response, meeting the accuracy requirements for the next generation of CMB experiments.

As a baseline option, the calibration satellite is based on the CubeSat standard carrying a perfectly known source able to emit a reference signal to reduce CMB polarization measurements uncertainty. CubeSat has become a satellite concept with increasing interest for space research and exploration. Key elements of its success have been standardization, easy design and low cost. Recent missions such as CanX-4 & 5 [12] have shown that CubeSats already have the necessary technology to carry out formation flight. Formation flying multiplies the possibilities of performing science with small satellites. As an example, the future ESA’s technology demonstration mission PROBA-3 [13] can be mentioned. In this mission, one of the satellites of the formation flight acts as a parasol, hiding the solar disk so that the other can study the solar corona. PROBA-3 is composed of two smallsats (less than 500 kg), but it is not a CubeSat mission. Nevertheless, a growing number of missions using formation flight are based on CubeSat technology [14].

The scientific scope of the CubeSat concept is limited by its constraints of space, weight and power, and the consequent impact in the scientific instrumentation that can be accommodated. However, it is expected to be a very competitive solution for scientific applications that can be adapted to those constraints, as the calibration of ultra-sensitive CMB-Sats operating at L2, like LiteBIRD [15,16], PICO [17] or CORE [18] (see also the microwave spectro-polarimetry white paper for the “Voyage 2050” long-term planning of the ESA science programme [19]). Although the calibration satellite concept can be adapted to the characteristics of any of those missions, the CubeSat designed in this work takes into account the main characteristics of a LiteBIRD-like small aperture CMB space mission. The proposed calibration satellite has been conceived to travel as a piggyback integrated on the service platform of the main ship. It would be deployed once in the L2 orbit, to start the communication with the CMB-Sat for the formation flying and calibration activities. Furthermore, the small weight and cost of the CubeSats would allow them to be added to the main mission without having a great impact, and also to be conceived as science enhancements of the CMB polarization mission without jeopardizing it.

According to the scanning strategy followed by the CMB telescope, the influence of the relative position between the two satellites in the calibration process has been studied. The relative motion of the spacecrafts has been considered with a simplified dynamic model. Based on the calibration requirements, the subsystems of L2-CalSat have been sized and preliminary designed to evaluate its feasibility. The design has been carried out under the principle of minimizing the impact on the main spacecraft architecture.

The paper is organised as follows. In Section 2 the calibration requirements of CMB polarization missions are analysed. Section 3 is dedicated to the impact of the proposed calibration satellite on different systematic errors. In Section 4 L2-CalSat is described while Section 5 is dedicated to its operation at L2. Finally, some conclusions are presented in Section 6.

## 2. Calibration Requirements

The information obtained from the intensity and the polarization of the CMB have provided a unique source for probing different cosmological scenarios and capital aspects of fundamental physics. The reasons is that the physical mechanisms that take place in the early Universe, and that affected the CMB radiation during its path to reach us, have left very weak but significant anisotropies on the otherwise uniform microwave background. Many properties of our Universe have been revealed (e.g., flatness of the space-time, age, and energy and matter content [20]). Electric-like (E) and magnetic-like (B) modes of polarization are distinguished by their behaviour under a parity transformation *n* →−*n*. The harmonic coefficients of the E-mode show ${\left(-1\right)}^{l}$ parity whereas the ones of the B-mode behave as ${\left(-1\right)}^{l+1}$. As a consequence, a parity transformation affects only the sign of B-modes but not E-modes. The Standard Cosmological Model (SCM) establishes that temperature and E-mode polarization anisotropies were originated by density perturbations while the B-modes are only sourced by tensor perturbations [21] (despite a contribution coming from E-modes due to gravitational lensing).

However, while recent polarization experiments are able to detect E-mode signals that supports the SCM [22], primordial B-mode signals have yet to be detected [23]. They are fainter and can be easily contaminated (in particular, by the lensing of the E-mode polarization and by astrophysical emissions from our Galaxy and extra-galactic sources) but they can provide essential information about inflation, the signal level of the primordial gravitational wave background (PGWB), the species and mass of neutrinos [24] or the magnetic fields, both primordial (PMF) and also those present in galaxies and galaxy clusters [25]. They may also be used to study other crucial aspects as cosmic birefringence (CB) [26,27,28], dark matter or extensions of General Relativity [18,19]. As mentioned above, polarization B-modes are sourced by different mechanisms, like the PGWB originated at inflation, primordial magnetic fields, the lensing of the CMB, or astrophysical foregrounds (notably dust and synchrotron emissions). Indeed, in 2014 the CMB community was attracted by the claim of a B-mode detection above the lensing level reported by the BICEP2 experiment [29]. This detection was afterwards explained by an accounted dust contamination [30], showing the important role that dealing with foregrounds contamination has in the study of the B-mode science. However, these genuine sky signals can be confused with spurious instrumental effects. In general, the spurious B-modes originating from polarization systematics cause a bias that hinders the extraction of cosmological information from the observed sky signal. For instance, the use of a continuously-rotating HWP coupled to fluctuations in the time constants of the detectors can lead to apparent shifts in the polarization angles of the detectors. Additionally, in-flight variations of detector responsivity with respect to the laboratory calibration as well as responsivity stability, also require a precise calibration during observation.

Moving forward, the high sensitivity levels required by current and future ground [31,32,33,34] and space-based CMB polarization experiments make that, systematic effects, previously only partially considered due to higher statistical uncertainties, now become the most significant limitation. Such experiments need an independent experimental calibration approach allowing one to trace systematics affecting, among others, to intensity, polarization and radiation pattern [11,35].

### Calibration Methods

Existing methods for calibrating CMB polarimeters [36] result in limited accuracy, among other aspects, on the determination of the polarization angle and the beam patterns. This is due to several reasons, being the lack of natural reference sources one of the most important limitations. The calibration of polarized beams (which requires a very well known and purely polarized signal source placed in the far field of the telescope) and polarization angle, need of frequency-independent and time-invariant point sources. Furthermore, they should be always visible independently on the site of the telescope. Actually, Tau-A is the best astronomical candidate for polarization calibration, but presents a limited polar angle accuracy between $1^{\circ }$ and $0.5^{\circ }$ [10].

On the other hand, microwave detectors must be cooled down from 300 K to 100 mK [37]. As a consequence, another source of inaccuracy is coming from very small contractions of the pieces in the cryostat, which could induce over 1mm misalignment, which represents the minimum uncertainty requested to reach the $1^{\circ }$ accuracy in the alignment that detectors and optics must satisfy. This accuracy is difficult to achieve, even if a specific design to reduce these effects is planed, since all the devices has to be mounted at room temperature, and, during operations, external agents (like pressure and temperature) can vary, affecting the cryostat conditions. Finally, and additional critical step is to relate the orientation of the detectors relative to the telescope and the receiver mount, once the cryostat is closed. This will imply, at the end, that it is not possible to have a direct polarization angle calibration with an accuracy better than $1^{\circ }$.

Additionally, several on-going and planed CMB experiments contemplate large rotating Half-Wave Plates (HWP) to act as polarization modulator systems [38]. This is a natural choice, since their large size allow them to determine the relative polarization angle with high accuracy, avoiding that edge effects could introduce, for instance, optical aberrations. However, in practice, actual HWPs present also non-idealities that can degrade the accuracy. Other calibration strategies are based on the use of polarization grids placed between the optics and polarimeters but, as a result, the don’t allow to characterize beam systematic errors provided by those optics.

Many experiments use a method called self-calibration [39] in order to overcome the lack of good astronomical calibrators. This method uses one prediction of the SCM to calibrate CMB polarimeters, which conclude that the TB and EB signals identically vanish due to the correlation absence between odd- and even-parity primordial signals. However, physical phenomena as CB could generate non-null TB and EB signals. In such a case, self-calibration prevents the study of these phenomena and introduces errors on the cosmological parameters.

For the particular case of CB, it has been recently shown [26] that it is possible to disentangle miscalibrated polarization angles from CB due to the fact that both effects rotate the CMB polarization angles but only the miscalibration angle rotates those of the Galactic foreground emission. While this result represents an improvement on our ability to constrain parity-violating physics with CMB polarization data, a precise calibration of the absolute polarization angle for the different frequency bands of a given experiment remains as the optimal solution for the scientific exploitation of the data.

In the case of previous CMB missions (Planck (ESA) [40] and WMAP (NASA) [41]) which presented a reduced number of detectors, celestial sources could be used for in-flight calibrations of the beam and the polar angle. As previously noted, for the polar angle, Tau-A is known to date with an accuracy of about $0.5^{\circ }$. For the beam characterization, Jupiter provides an unpolarized thermal signal allowing a noise floor (NF) level of about −50 dB at 100 GHz [40,42], taking into account the overall contributions of this particular detectors band during the complete observation period of a typical mission. However, for future missions with thousands of detectors the level of accuracy reported by these sources seems clearly insufficient to extract all the potential scientific results expected from them.

## 3. Calibration Mission Impact over Systematic Errors

In this section, the impact of the proposed calibration system is analysed over three very important systematics, that can be clearly benefited by a calibration satellite : the polarization angle, pointing and beam pattern. For the optimal calibration of these effects, a point-like source should be viewed in the far-field of the telescope (see Table 1 for some example distances).

While other systematic errors could be corrected in-flight without the requirement of an external source, however, it is important to note that the proposed calibration system allows also to characterise all those additional errors (gain, non-linearity, spectral or band-pass response, etc.) and takes into account the complete optical path of the CMB polarimeters. Thanks to the accurately characterized artificial source embarked in L2-CalSat, the calibration of fully integrated and cold receivers will be possible, allowing the characterization of any misalignment due to thermal contractions in operation at L2. Additionally, systematic errors from optical or mechanical systems (HWP, filters, etc.) can be characterized, taking into account the complete optical path. Note again, that by using the proposed calibration system, the assumptions on TB and EB signals will not be required, allowing an accurate determination of cosmological parameters and the measurement of CB, between others physical phenomena.

The statistical characteristics of the CMB anisotropies are usually studied by means of the angular power spectrum. The variance of the spherical harmonic coefficients from temperature (TT) and polarization (EE, BB) fluctuations are represented, as a function of the multipoles l. Additionally, the correlation between temperature and polarization are also represented by the cross spectra TE, TB and EB. Overall, spurious polarization effects produce a power leakage from the T- and E-mode signals that result in a systematic error in the BB, TB and EB spectra.

### 3.1. Polarization Angle

**Figure 2:**
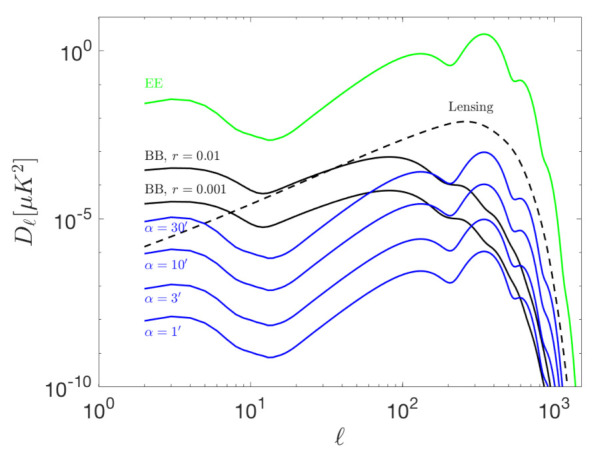
Systematic errors in the BB angular power spectra per logarithmic interval produced by polarization rotation. The spurious signal produced by 30, 10, 3 and 1 arc-minutes of rotation are represented by the blue curves. L2-CalSat would allow the suppression of any polarization angle errors to the region below the 1 arc-minute blue curve. Primordial B-mode for two values of *r* (solid black lines), gravitational lensing B-mode (dashed black) and the EE (solid green) spectra are plotted for reference. A Gaussian beam of FWHM = 30 arcmin has been adopted.

**Figure 3:**
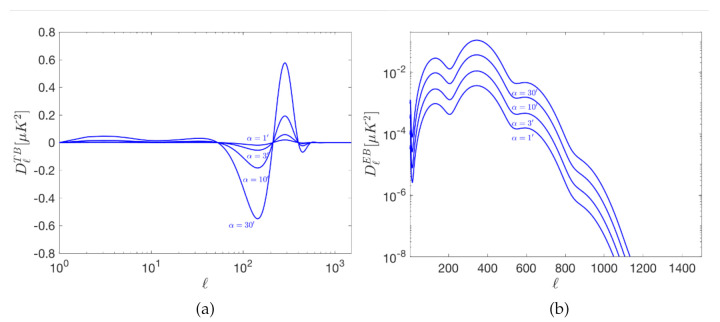
Systematic errors in the TB (**a**) and EB (**b**) angular power spectra per logarithmic interval produced by polarization angle errors. The spurious signal produced by 30, 10, 3 and 1 arc-minutes of polarization rotation are represented by the blue curves. The same assumptions of Figure 2 have been adopted.

As previously explained, systematic errors in the measurement of the polarization angle produces a leakage from the E- to the B-mode signal. Additionally, a correlation between them is introduced which is reflected in non-vanishing TB and EB signals. Figure 2 and Figure 3 show the systematic errors in the BB, TB and EB power spectra produced by a polarization angle rotation. These results have been obtained from the expected leakage for a given polarization angle mismatch, under the assumption that the angle error is common to all detectors.

Blue curves represent the spurious signal produced by 30, 10, 3 and 1 arc-minutes of polarization rotation in both figures. Primordial B-mode (solid black lines), gravitational lensing B-mode (dashed black) and the EE (solid green) signals are also plotted for reference in Figure 2. It is important to note that 30 arc-minutes ($0.5^{\circ }$) of rotation is approximately the current error level set by the systematic uncertainty of celestial calibration sources as Tau-A. The calibration sources of L2-CalSat would allow the reduction of any polar angle error below the 1 arc-minute blue curve. This error is sufficiently low for current and future experiments, both in the ground (like the CMB Stage IV [31]) and from space (like LiteBIRD) that aim at a sensitivity in r of around 0.001. Notice that if the correction is not made with enough accuracy, this will bias the detection of the B-mode, mostly at the recombination peak.

### 3.2. Pointing

**Figure 4:**
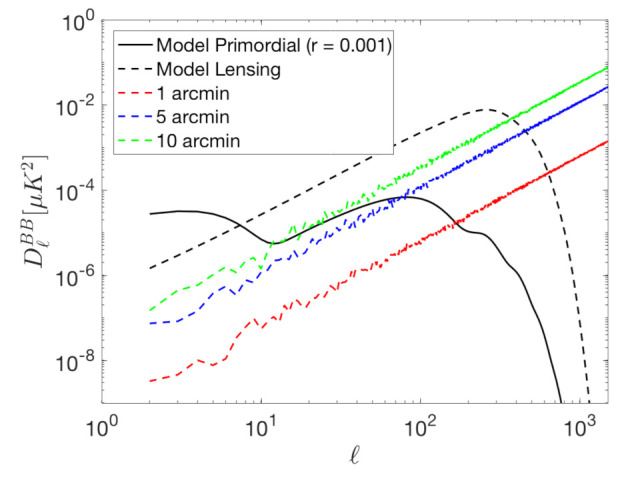
BB signals produced by random pointing errors of 10 (green), 5 (blue) and 1 (red) arc-minute.

**Figure 5:**
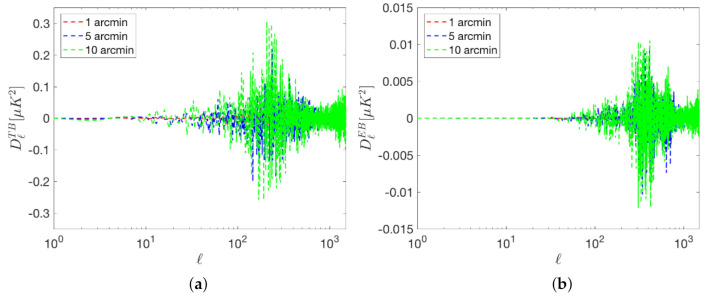
TB (**a**) and EB (**b**) signals produced by random pointing errors of 10 (green), 5 (blue) and 1 (red) arc-minute.

Pointing errors can also produce TB, EB and BB spurious signals. For instance, focal plane misalignment can lead to differential pointing errors between the detectors, resulting in T to B leakage, especially on small angular scales. Figure 4 and Figure 5 shows the simulated leakage signals for random pointing errors of 1, 5 and 10 arc-minutes. This effect is simulated by remapping each pixel of the CMB maps (T, Q and U) into a new pixel at a distance randomly Gaussian distributed, with a dispersion given by the above-mentioned pointing errors, and towards a direction randomly selected according to a uniform distribution. In Figure 4, it can be observed that this kind of errors are specially problematic in the case of BB signals at very high *ℓ*. In the TB and EB spectra of Figure 5 it can be observed that random pointing errors affect mainly at multipoles above ℓ∼100. As for the case of the systematic associated to the polarization angle discussed in the previous subsection, this effects, if not properly accounted for, biases the B-mode detection, in particular, at the recombination peak.

### 3.3. Beam Patterns

Important polarization systematics can be produced by the leakage from intensity to polarization signals, due to non-idealities in the beam patterns of the telescopes, if they are not well understood and accounted for. Beam patterns are a 2D representation of the angular response of the telescope. Precise knowledge of them is crucial to reconstruct the sky maps and extract accurate scientific results. As in the previous cases, beam irregularities can produce TB, EB and especially BB spurious signals. They arise from different effects related to the optics, the filters, the focal plane, and the readout electronics and can be quantified and taken into account properly, only if characterised during the operation of the telescope.

The best method to measure beam patterns is to observe a well-characterized point-source in the far-field of the telescope. Ideal beam patterns should be symmetric and should not affect the polarized properties of the incoming light. However, they often exhibit different properties for different detectors and their shape can be affected by ellipticity and other irregularities. These effects introduce polarization systematics and polarization leakage, including non-zero TB and EB signals, and, more importantly, BB bias. Sometimes beam features can be taken into account with optical models, but some complex features must be quantified using an experimental beam calibration. Examples include features caused by stray light from internal reflections or scattering on optical elements, mirror surface errors, diffraction, transmission elements emission, and non-uniformities.

Beam window functions are also affected by focal plane systematics, such as feed-horn asymmetries or cross-polar response, detector time constants, and detector orientation. Coupling with sinuous antennas and lenslets may introduce cross-polarization and beam ellipticity as well. Sinuous antennas have a periodic, frequency-dependent polarization angle defined by the antenna geometry.

**Figure 6:**
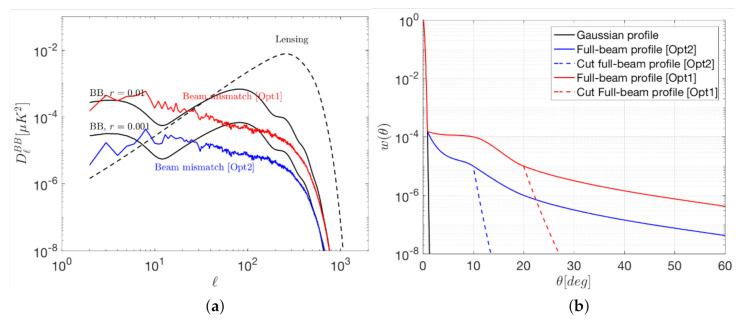
BB signals (**a**) produced by the FSL of two non-ideal beam models (**b**) with FWHM = 30 arcmin and for a frequency of 140 GHz. Red lines (option 1): spurious signal produced by FSL placed at more than $20^{\circ }$ from the beam center. Blue lines (option 2): spurious signal produced by FSL placed at more than $10^{\circ }$ from the beam center. Primordial B-mode (solid black lines) for two values of *r* and gravitational lensing B-mode (dashed black) spectra are also plotted in (**a**) for reference.

In order to accurately characterise the intensity beam, the Planck [40,42] CMB space mission showed that with several years of observations of a typical celestial source as Jupiter, a NF level of about −50 dB respect to the peak amplitude of the planet emission can be achieved, taking into account the overall contribution of the cosmological bands detectors. Although this level of accuracy can be slightly improved combining the data from other planets as Mars, Saturn, Uranus and Neptune, in the case of future ultra-sensitive space missions, it may result insufficient to characterise far side-lobes (FSL) placed at $10^{\circ }$ or more from the beam center, specially in polarization. To illustrate this, Figure 6 shows the BB signals (a) produced by the FSL of two non-ideal beam models (b) with FWHM = 30 arcmin and for a frequency of 140 GHz. The beam coloured in red has been modeled following the trend of the ones shown in [43], while the beam coloured in blue has been modeled from the red one but presenting 10 dB lower side lobes from $10^{\circ }$ onwards. In this case, the spurious signal is produced by FSL placed at more than $20^{\circ }$ from the beam center, which would remain non-characterised using Jupiter. It has been calculated subtracting the signal of an auxiliary beam that is equal to the red one until $20^{\circ }$, but from this angle it is equivalent to a Gaussian one (see the dashed red line in Figure 6b). In the case of the beam coloured in blue the spurious signal is produced by FSL placed at more than $10^{\circ }$ from the beam center and it is calculated, equivalently to the red one, using an auxiliary beam that behaves as a Gaussian one from $10^{\circ }$ onwards. For such a beam, Jupiter could provide information only until this angle, remaining the rest of it non-characterised. However, due to its more selective profile the produced spurious signal is about one order of magnitude lower than in the previous (red) case. For both beams it has been considered a reference NF level of −50 dB (available using Jupiter), which would allow to characterise them until $10^{\circ }$ (blue) and $20^{\circ }$ (red) from the beam center. However, at larger angles powerful signals coming from the galaxy could contaminate the CMB observations. It can be observed that, targeting r=0.001, the spurious BB signal produced by the beam in red would mask the primordial signal for almost all multipole values while the beam in blue would be also an issue for multipole values around 10.

## 4. L2-CalSat Description

In this work it is proposed the use of a calibration source embarked on an ancillary satellite (L2-CalSat) that would be in formation flying with the main CMB scientific satellite (CMB-Sat). As will be shown in Section 4.4, the unpolarised thermal emission of the calibration satellite could saturate the highest frequency band detectors on-board CMB-Sat, even when placed at the far field distances of Table 1. So, in order to avoid this issue, we propose to place L2-CalSat at the necessary distance from CMB-Sat, which results to be about one order of magnitude greater than the one shown in Table 1. For small aperture experiments (LiteBIRD), this calibration system will enable control over pointing errors and polarization systematics, allowing the calibration of the polarization angle, far field measurements of the telescope beam patterns, and the calibration of the intensity response. For larger apertures (CORE or PICO), the larger distances required to reduce thermal emissions from the calibration satellite, makes harder the achievement of the required calibration signal power level, and also the orientation accuracy.

However, in such cases, it would be also possible to use the proposed calibration system by adding to L2-CalSat some kind of cooling system (for shorter distances between satellites) or a thermal calibration source able to provide more power at the highest frequency bands (for larger distances). In any case, this paper is focused in small-aperture experiments that can be calibrated with a simpler system, letting the case of larger aperture missions for a future work.

In the proposed calibration system a set of microwave sources will emit linearly polarized signals at different wavelengths, covering the frequency bands of the CMB-Sat polarimeters, typically between 40 and 700 GHz. The calibration satellite will operate from far field distances, providing:

- Narrow band signals with high signal to noise ratios, to explore in-band features of the optical elements.
- Wide band signals, to calibrate the integrated bands of the instruments with a signal similar to that received from the sky.
- Polarized signals, used primarily to calibrate the absolute polarization angle, but also to measure beam systematics and for intensity calibration.

L2-CalSat will fly in formation with the main satellite, appearing as a distant source for the polarization sensitive detectors. It is important to note that the developments and results of the ESA technology demonstration mission PROBA-3, to be launched in 2022, will represent a clear benefit for the accurate formation flying required for L2-CalSat.

Our proposed calibration mission, far from the Earth, is very challenging because it involves formation flying without support from a GPS navigation system. Therefore, the Attitude and Orbit Control System (AOCS) has to provide not only support for the orientation of the calibration payload (source) but also navigation around the main satellite. As calibration system, the AOCS will be used to position the source and to register the polarized source direction to in sky coordinates. Two celestial coordinates (from an accurate star camera pointing direction) will be used to determine the third angle defining the rotation of the polarization plane along the line of sight of the detectors. In the absence of a GPS system, and also for security reasons, the main satellite should provide navigation information for L2-CalSat, the latter requiring also a propulsion system. On the other hand, a clear advantage of orbiting L2 instead of Earth comes from its lower risk in terms of micrometeorite bombing and, in particular, of space debris coming from the increasing number of satellites in that region of space. In this sense, the risk of impact can be calculated using models that estimate the background meteoroid flux (continuous and from certain directions of space) according to its mass. In addition to the influx of background meteoroids, there are quasiperiodic events caused by streams of materials from comets that pass close to Earth and therefore near L2. Other kind of threats as, for instance, solar wind could also affect to both, the navigation instrumentation and the payload stability. Previous missions in L2 as Gaia and Herschel have shown that the radiation environment can be considered similar to that of GEO and high inclination LEO orbits or even more benign in some aspects (trapped particles are avoided, for example). Although single events are more prone in this region, specially during periods of high solar activity, protections measures can be implemented in both software and hardware. Nevertheless, the technology of the navigation instrumentation and the payload has been used in previous missions (flying far from Earth like MarCO [44] and CMB missions like Planck, respectively) without detecting particular issues from that aspect, thanks to the shielding systems implemented on them.

In order to study the general stability performance of L2-CalSat, in principle it is not considered the application of long-term vacuum chamber tests at space temperature because the instrumentation will be operating at a temperature close to the usual ambient one (typically between 0 and 50 Celsius, as will be seen later) thanks to the incident radiation coming from the Sun. On the other hand, previous experiences tell us that the operation of the instrumentation (both, navigation and payload) it is not affected by the fact of being in vacuum conditions. However, other laboratory tests are required to characterise the gain and, consequently, emitted power variations that can happen due to expected temperature changes in the previously referred range (0–50 Celsius). This kind of tests are performed using temperature chambers that are easily available and require only hours or days for a complete and accurate characterization.

Due to the large technological development carried out during the last years and the low-cost of CubeSat platforms and Commercial off-the-shelf (COTS) components, we have studied their suitability for the L2-CalSat platform. In particular, a 6U CubeSat is expected to meet the volume needs of L2-CalSat. It would travel as a piggy-back of the CMB-Sat which, until the deployment in L2, must provide structural support and a minimum power supply to maintain the batteries of L2-CalSat. Once it is deployed, some hardware would remain attached to CMB-Sat for communication and navigation support tasks. These subsystems would operate as autonomously as possible, with their own solar panel and batteries. Nevertheless, it would be necessary a minimum capacity of communication with CMB-Sat for calibration synchronization and downloading data to ground. Following the CubeSat and COTS philosophy, there exist commercial separation systems, which fulfil the requirements. Other implementation option for L2-CalSat would be a micro-satellite capable of reaching L2 autonomously from the same launcher. As the thrust required in L2 is very small (the orbit presents low-force gradients), one of the main differences with the piggy-back option would be the required propellant, taking into account that only the transfer to L2 would require an impulse (Δν) of about 290 m/s, in comparison with the 17 m/s (see Section 5 ) of total Δν required in the base-line (piggy-back) option. The micro-satellite option would be very flexible at system design level, but its cost would be significantly higher, limiting so the viability of the concept in terms of practical implementation.

### 4.1. Requirements

Focusing on small aperture CMB missions, the following general requirements have been established, based on formation flight of existing missions, previous experience, and thermal considerations (see Section 4.4):

1. L2-CalSat shall be around L2 using a Lissajous orbit and maintain that orbit during 3 years.
2. L2-CalSat shall maintain a minimum-security distance of 4620 m from the main satellite and a distance of about 6600 m ± 66 m during, at least, 6 h for each calibration session.
3. The maximum time to move away L2-CalSat from the main satellite FoV shall be less than 20 h.

On the other hand, in order to achieve the required polar angle and beam characterization accuracy (see Section 3.1 and  Section 3.3), the following technological requirements related to the AOCS, Electrical Power System (EPS), payload, propulsion and structure, have also been considered:

1. The sensors shall locate the direction of the main satellite with an error lower than 10 arcmin and shall know L2-CalSat orientation with an error lower than 1 arcmin.
2. L2-CalSat shall point to the telescope with an error less than 3 arcmin.
3. EPS shall fulfil every power subsystem requirement and provide enough energy to the payload to allow, at least, 2 calibrations per year. It has been considered a maximum power consumption of 50 W during calibration.
4. During calibration, the sensors shall be able to calculate the distance between both satellites with an accuracy of 3.3 m.
5. The propulsion system shall be able to provide enough thrust to fulfil the calibration requirements while providing a displacement error of less than 3.3 m.
6. L2-CalSat dimensions shall fit in a 6U CubeSat.
7. L2-CalSat must have minimum impact on the main CMB satellite.

### 4.2. L2-CalSat Design

**Figure 7:**
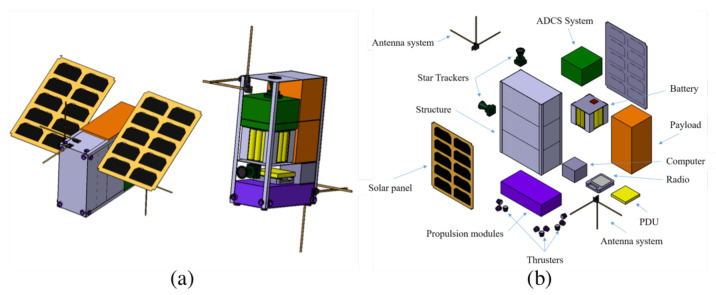
Exterior view (**a**) and exploded view (**b**) of the preliminary design for L2-CalSat. ADCS is the Attitude Determination and Control System, referred as AOCS in the text. PDU is the Power Distribution Unit.

To obtain a first design of the calibration satellite from the requirements shown previously, a session has been carried out in the IDR-UPM’s Concurrent Design Facility (CDF). Figure 7 shows the exterior view (a) and exploded view (b) of the resulting 6U cubesat.

From the subsystems shown in the previous figure, those that represent critical technologies have been identified as the following:

1. For the attitude control and determination, Fine Sun Sensors by GomSpace [45] and star trackers by CubeSpace CubeStar [46] can be used.
2. For metrology (relative position determination) and communications, RF ranging, a flight-proven technology  [47] able to provide the required accuracy. Tethers Unlimited has developed an integrated system for cubesats [48] although it is also possible to build the system with COTS [49] (antennas, On-Board Computer (OBC) and software-defined radio).
3. For the propulsion of the CubeSat, cold gas thrusters to provide small and precise impulses with low consumption, like GOMSPACE NanoProp [50], can also be used.

#### Payload, Communications and OBC

The main payload of L2-CalSat is a multi-frequency variable source, covering the overall bandwidth of the particular CMB mission to be calibrated. Both, the signal frequency and power should be variable in order to characterise completely systematic errors of the instrumentation on-board CMB-Sat. The power level has to be optimised for the detectors to be working in their nominal range, avoiding saturation. Due to the wide frequency-range to be covered, it is necessary to implement a multi-frequency source, generating monochromatic amplitude- and frequency-variable signals and also broadband signals, covering several sub-bands determined by the commercial wave-guide standards. The calibration source is described in detail in the next subsection.

Two additional subsystems are considered as secondary payload. The first one is a communication system to determine the distance between L2-CalSat and CMB-Sat, and also the relative position when calibrating. The second subsystem is an On-Board Computer (OBC), which must be able to control, as efficiently as possible, the main payload and the communication system.

This mission requires very accurate knowledge of relative positioning and has very strict orientation requirements during calibration. In order to determine the distance of the telescope and the relative position with the desired accuracy, it is proposed the use of a radio-frequency communication module, recently established for the formation flying mode of the SULFRO [51] concept. In particular, it is proposed an inter-satellite communication system (like Swift RelNav) based on a pair of communication modules, each one with two sets of three orthogonal dipole antenna and a radio transceiver. Notice that one module is placed in L2-CalSat and another module must be placed on the main satellite. The configuration is depicted in Figure 7.

In addition, an OBC and a Software Defined Radio (SDR) algorithm will be also required. The SDR algorithm is needed for distance measurement and orientation determination during calibration, to make sure that both satellites are maintaining their optimal configuration of relative position. On the other hand, the OBC is required to process the information coming from the array of sensors, and engage the reaction wheels accordingly. Besides, the OBC must be mounted onto a dock board in compliance with the PC104 standard.

The OBC module NanoMind Z7000 [52] designed by GomSpace has been finally selected, because it is able to process a wide range of operations and calculations to determine the relative position and distance of the telescope, while fitting with the strategy of using cost-effective devices. In addition, this OBC has already been used in several CubeSat missions presenting good performance.

### 4.3. Calibration Sources

Focusing in a frequency band from about 40 to 400 GHz, and trying to optimise volume, cost and power consumption, it is proposed to cover six bands determined by the corresponding wave-guide standards: 33–50 GHz (WR22), 60–90 GHz (WR12), 90–120 GHz (WR8), 140–220 GHz (WR5), 220–325 GHz (WR3) and 325–500 GHz (WR2). Note that the 50–60 and 120–140 GHz bands are not covered by the sources. For simplicity, we have assumed that they can be omitted in this first study but, if necessary, an alternative frequency coverage could be considered in a future analysis. It is critical to include the higher (WR2) frequency band since at these frequencies it is not possible to use the orbital dipole (a Doppler effect generated by the orbital motion of the CMB mission) to calibrate the intensity, as was done by the Planck mission for lower frequencies.

**Figure 8:**
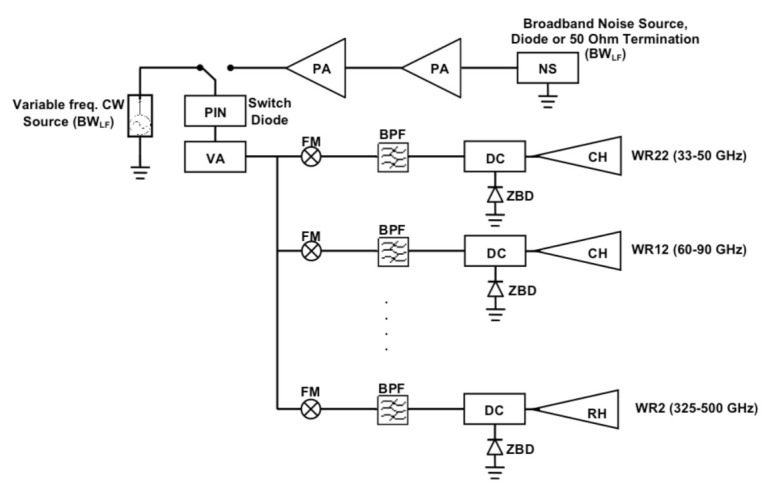
Diagram of a calibration source covering frequency bands from 40 to 400 GHz.

In order to generate calibration signals covering all these frequency bands, it is proposed the use of two common low-frequency sources (monochromatic frequency-variable and broadband) that can be used to generate variable signals. Then, the required bandwidth coverage will be achieved by means of power splitters and Frequency Multipliers (FM) as those supplied by, for example, Virginia Diodes [53] (USA), Viva Tech [54] (France) or Farran Tech. [55] (Ireland). Figure 8 shows a schematic of this kind of source implementation.

**Figure 9:**
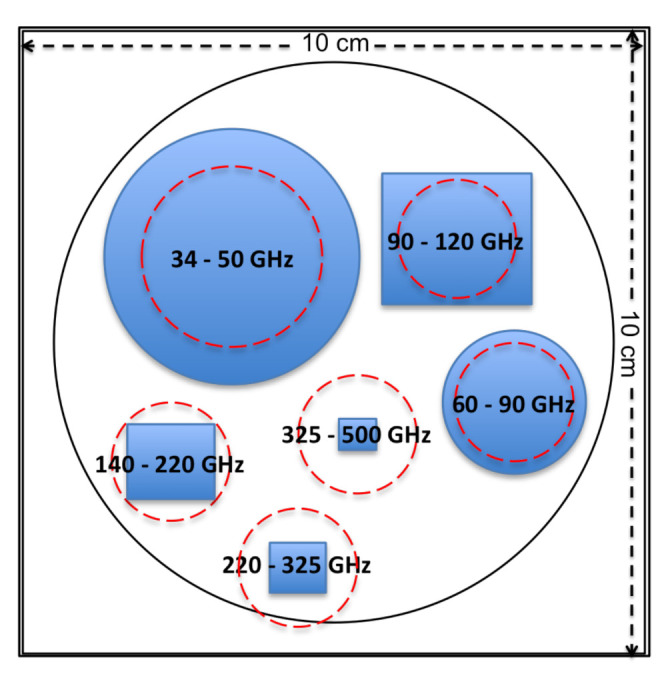
Frontal view of the flying calibration source showing the feed-horns sizes (in blue) and the corresponding wave-guide flanges (red lines), which determine the size envelope at the highest frequencies.

It consists of a variable low-frequency Continuous Wave (CW) source, which is able to cover a given bandwidth $BW_{LF}$, and a broadband noise source (NS) covering the same bandwidth, feeding both of them a common frequency multiplying system that translates the signals to the required frequency bands. The broadband NS can be implemented by means of a noisy diode or a 50-Ohm termination connected to a Power Amplification (PA) chain with the same bandwidth $BW_{LF}$ that will provide the required level of signal power. A PIN (P-type, Intrinsic semiconductor, N-type) switch diode is used to select the kind of signal to be used, as a function of the particular systematic to be calibrated. A variable attenuator (VA) is used to change the power level for linearity and beam calibrations. VA applied directly to the common low-frequency signals will reduce the cost and complexity of the overall source. The signal is then divided to feed the sub-band transmitters and the frequency of the signal is multiplied using FM to get the required frequency coverage in each of them. A Band-Pass Filter (BPF) rejects undesired harmonics of the signal and a Directional Coupler (DC) is used to measure the emitted power level by connecting a Zero-Bias-Detector (ZBD) to the coupled output. The direct output is connected to a feed-horn providing the calibration signal of each sub-band. Due to the difficulties to generate microwave power at the highest frequencies, it has been proposed the use of conical horns (CH), with 20 dB gain, for the two lower frequencies bands and rectangular horns (RH), with 26 dB gain, for the rest of bands. Figure 9 shows a sketch of the frontal view of the calibration source mounted in a 2U CubeSat chassis of size 10 × 10 × 20 cm.

Another requirement of the calibration source is to provide a very pure linearly polarized signal, presenting a cross-polarization degree of the order of −60 dB. This can be also achieved by means of the use of Wire-Grid Polarisers (WGP) placed in front of the source’s horns and accurately aligned with their polarization axes [11,35].

Table 2 summarises the final main payload physical characteristics.

### 4.4. Thermal Control and Power Budget

The thermal design of L2-CalSat will maintain the payload within a range of temperatures in order to meet survival and operational requirements during all mission phases, which are to be established to ensure the stability of the payload. In order to validate the design and characterize the performance of the payload under thermal loads during its operation, functional tests and thermal cycle tests (rapid changes between hot and cold temperatures to stress the hardware) will be carried out. A preliminary thermal design of the calibration satellite has been implemented in the ESATAN^®^ software. Model simulation shows that a maximum temperature of 39 ${}^{\circ }$C is reached in part of the surface of the solar panels that is facing the sun. In order to be conservative, this temperature value has been set for the complete surface of the panels, and the corresponding thermal radiation reaching the focal plane at the frequencies of interest has been calculated. Table 3 shows the maximum power of the calibration signal from L2-CalSat (second column) compared with the thermal power arriving from Jupiter (fourth column) to the focal plane, for 4 frequency bands required to characterise the synchrotron (40 GHz), CMB (100 and 200 GHz) and thermal dust (400 GHz) emissions. In order to reduce the power consumption of the payload, except for the 40 GHz frequency band, passive frequency multipliers, which provide a limited power level, have been considered. Additionally, in the third column, the unpolarized thermal power arriving from the calibration satellite in the worst-case scenario (the surface of the solar panels is facing perfectly the telescope aperture), is shown. It is important to note that, considering an uncontrolled tilting angle of L2-CalSat of 3 arc-minutes, the resulting thermal power variation is not considered to be an issue because it represents only about a 0.004 % of the values in Table 3.

On the other hand, Table 3 also shows that the thermal signal from L2-CalSat is always lower than the one from Jupiter, considering a distance of 6600 m between both satellites. In order to check the incidence of this signal over the detection stage, the last column of Table 3 shows the power from CMB and the saturation power level of each detector [56]. It can be observed that the thermal power from the satellite is below the saturation power of the detectors, even for the highest frequency band (400 GHz).

Finally, it is also important to take into account the effect of the reflected signals from the solar panels, because this signal is perfectly (100 %) polarized in the previously cited worst-case scenario. However, the power level of that kind of signal has been calculated in those conditions, resulting about four orders of magnitude lower than the thermal emission from the panels, at all frequencies. Thus, these reflected signals have been discarded as a source of interference, as in the worst case (400 GHz) it would be three orders of magnitude below the CMB signal.

### 4.5. Overall Features and Implementation Options

Taking into account all the previous information, the overall characteristics of the preliminary design for L2-CalSat are summarised here. The calibration satellite total mass will be 7.193 kg (7.07 Kg of dry mass and 0.123 kg of propellant). The required electrical power will be provided by 2 orientable 6U-CubeSat solar panels with triple-junction solar cells by SpectroLabs and accumulated in a battery module (see Figure 7). The solar panels will continuously generate 30 W, enough to feed all the subsystems of L2-CalSat and charge the battery. During calibration, the solar panels and battery combined will provide a peak power of 62 W to supply the payload and the rest of subsystems.

The final main characteristics of L2-CalSat are sumarised in Table 4.

The calibration system proposed here could reduce the uncertainty in polar angle to 1 arc-minute ($0.017^{\circ }$) due to the accurate alignment [11] of the source components with the structure of L2-CalSat and the precision provided by its ranging and pointing systems. Additionally, as can be seen from Table 3, it would provide a polarized calibration signal allowing a noise floor level reduction of about −22 dB, in the 100 GHz band, respect to the values achieved by using Jupiter as a thermal source, taking as a reference the values achieved by the Planck mission [40]. In fact, the next section will show that the calibration satellite could provide noise floor levels lower than −70 dB in the overall band of example (from 40 to 400 GHz), assuring so the complete and accurate characterization of the beam and in particular of the far side-lobes. With this level of systematic errors characterization, future CMB missions will be able to reach easily values of 0.001 in the tensor-to-scalar ratio *r*, which is a fundamental requirement in all of them.

Let us finally remark that if the piggy-back implementation option were not viable, the concept of L2-CalSat could be extended to a micro-satellite capable of reaching L2 autonomously from the same launcher. The modification would affect mainly to the propulsion and communication subsystems. The propulsion subsystem would be composed of two different types of thrusters, monopropellant (Hydrazine) and MEMS ($H_{2}O_{2}$). The communication subsystem should be capable of establishing contact with Earth by itself, unlike in the CubeSat implementation option where it has been assumed that the CMB-Sat should implement the communication interface between Earth and the calibration satellite. Therefore, an X-band high gain antenna would be required in the micro-satellite implementation option. The micro-satellite mass is limited to 100 kg of which roughly 13 % would be allocated for propellant, according to the propulsion subsystem specifications. In any case, this last option would have a significantly higher cost than the CubeSat scenario and should be programmed to be launched in the same rocket (or at least in the same period of time) than the main CMB telescope. So, it would be important to take all these factors into account from the beginning of the particular scientific mission design.

## 5. L2-CalSat Operation

As it has been previously explained, the proposed calibration satellite would travel as a piggy-back of the main satellite, to be deployed once in the required Lissajous orbit around the L2 libration point of the Sun-Earth system -see [57] for more details-. One of the main characteristics of this kind of orbit is the small amount of propellant needed to maintain the satellite on it. In fact, the total thrust required for a 3-year calibration mission has been estimated as Δν = 17 m/s, which is around one order of magnitude lower than that required to transfer a micro-satellite to L2.

After separation, the platform of the main satellite will be the communication link with L2-CalSat. The CMB-Sat will also need to take part in the AOCS of L2-CalSat, providing position and attitude references to calculate relative distances and orientations. The calibration system requirements for pointing at CMB-Sat have been considered, resulting critical the determination of the distance between satellites and the availability of a collision avoidance manoeuvre, although this risk can be estimated as very unlikely. The calibration satellite shall maintain a distance of 6600±66 m during operation, in order to avoid the saturation of the CMB mission detectors due to its thermal emission, while being in the far-field of the telescope at the highest frequencies of the experiment (450 GHz, in the particular example described in this work). Additionally, for security reasons it has been established a distance range between both satellites from 4620 to 8580  m (considered calibration distance plus or minus a 30%).

In-flight calibration must be performed periodically. During this phase L2-CalSat has to be re-positioned and controlled with precision, and a communication link with the main satellite has to be maintained. The CMB-Sat will eventually point to L2-CalSat and will receive the calibration signals. The number, duration and characteristics of these calibration operations have been analysed and the most convenient strategies are proposed in this section.

### 5.1. Calibration Strategy

**Figure 10:**
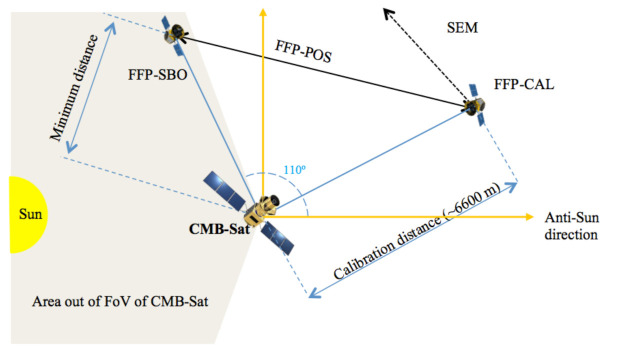
Diagram of the relative locations between the CMB satellite and L2-CalSat during the different phases of the mission.

In order to describe the calibration, first, it is needed to define the positioning maneuvers. L2-CalSat must be inside the FoV of the CMB-Sat during the calibration time and move away at finishing. Consequently, four impulses are required for each calibration manoeuvre (two to start movement and two to stop the calibration satellite). Figure 10 shows a diagram of the relative locations between L2-CalSat and CMB-Sat during the different phases of the mission.

Three mission phases are considered and constitute the timeline of the mission and its operations. The main of them is the Formation Flying Phase (FFP) that is active once L2-CalSat is in the vicinity of the CMB-Sat, after its deployment from the service platform. As it has been previously noted, the calibration satellite is expected to be equipped with RF ranging sensors, allowing the reconnaissance of the main spacecraft, and also the accurate determination of the relative position between both satellites.

In this FFP, three sub-phases are defined. The first of them is the Stand-by orbit (FFP-SBO). During the periods between calibrations, L2-CalSat will be in a waiting orbit, out of the field of view of CMB-Sat. In this phase, the relative position of the satellites will be maintained with a loose accuracy. The range of variation of the relative distance will be defined in order to minimize the need of propellant, while ensuring safety of both spacecrafts and a quick transition to the Calibration phase (FFP-CAL). In this second sub-phase, L2-CalSat is accurately placed with respect to a previously defined reference position of CMB-Sat, and maintained in a relative position and orientation, enabling L2-CalSat to periodically enter in the FoV of CMB-Sat. These periods are used for calibration of the CMB-Sat instrumentation. The calibration satellite design will ensure metre and arcmin relative position accuracy, which guarantees a proper a priory knowledge of the received signal, provided that the calibration payload constantly measures its output power. These measurements, properly time-stamped, together with AOCS and housekeeping information, will be transmitted to CMB-Sat which will send them to the ground for post-processing and calibration of the scientific data. The last Formation Flying sub-phase is the Positioning (FFP-POS). This is a transition phase in which L2-CalSat either enters FFP-CAL from FFP-SBO, or abandons FFP-CAL returning to FFP-SBO. This phase is entered either by ground command or by a timed-tagged or orbit-driven event. The manoeuvres needed for permitting transferring from FFP-SBO point to FFP-POS point and vice versa, will be optimized taking into account the specific L2 environment.

Additionally to FFP, in every phase of the mission, a Safe Escape Maneouvre (SEM) will be available. The design of Fault Detection, Isolation and Recovery (FDIR) of L2-CalSat will enable constant monitoring of the ranging sensors and the health status of the communications. In case of failure of any of them, or whenever a close approach to CMB-Sat is detected, a SEM will be triggered by making use of the remaining active positioning systems. The propulsion module will be commanded by the on-board computer (OBC) in order to provide a pre-determined thrust in a safe direction, thus avoiding collision with CMB-Sat or interference with its measurements. This manoeuvre will also be reachable by ground command.

Finally, the Disposal phase will ensure reaching a final orbit diverging from the one of CMB-Sat, so that avoidance of collision is ensured even beyond the lifetime of the satellites. This phase will be reached by ground command, either following the end of the useful life of the satellite, or upon determination of a non-recoverable failure.

### 5.2. Access Time and Viewed Detectors

**Figure 11:**
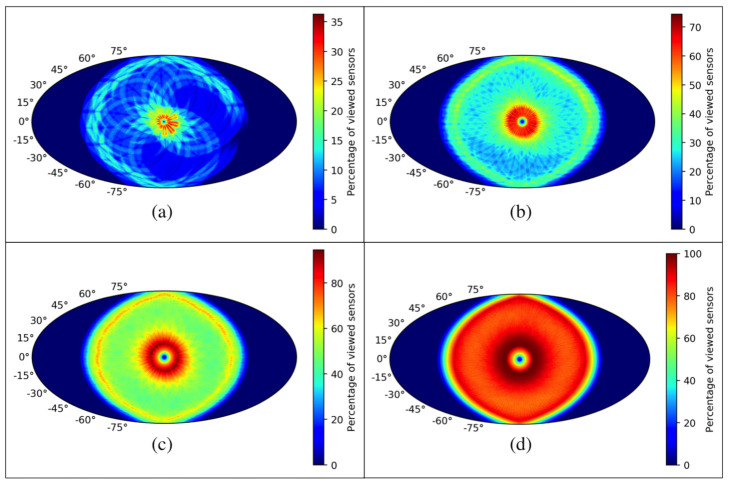
Percentage of viewed detectors according to the position of L2-CalSat relative to the CMB-Sat throughout 1.5 (**a**), 6 (**b**), 12 (**c**) and 24 h (**d**).

The scanning strategy of CMB-Sat and the L2-CalSat relative position, define both, the total access time and the number of viewed detectors. The number of detectors illuminated by the calibration signal has been determined by simulation, considering different relative position between both satellites. It has been noted that the more separated the different calibration signal traces are, the greater the percentage of viewed detectors. It has also been seen that the best option to place L2-CalSat is close to the anti-Sun axis, about $10^{\circ }$–$15^{\circ }$ from it. Figure 11 shows the percentage of viewed detectors according to the relative position between the two satellites throughout 1.5 (a), 6 (b), 12 (c) and 24 h (d) and considering a CMB-Sat FoV of $30^{\circ }$.

The percentage of viewed sensors (or detectors) is around 70% for 6 h (red region of Figure 11b) but it approaches 100% in that region for 12 h even when L2-CalSat is kept at the same position. It can also be observed that during a calibration session of 24 h, all the detectors could be covered from many more different positions; however, a calibration session of only 12 h has been assumed as baseline, since this calibration time would allow, among others, the polar angle calibration of almost the complete set of detectors.

**Figure 12:**
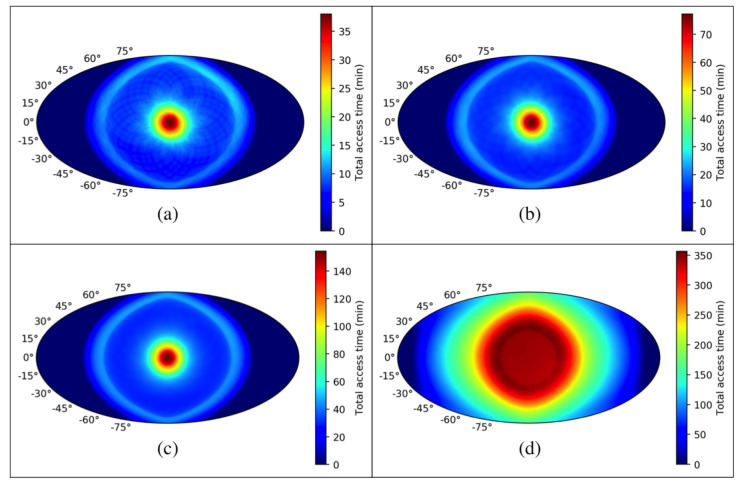
Total access time according to the position of L2-CalSat relative to the CMB satellite for a FoV of $30^{\circ }$ and throughout 6 (**a**), 12 (**b**) and 24 h (**c**), and for a FoV of $120^{\circ }$ and throughout 12 h (**d**).

The total access time is proportional to the number of accesses and how the signal crosses the focal plane. If L2-CalSat has a right ascension higher than roughly $15^{\circ }$ the waiting time between accesses would be equal to one precession period. Note also that the average access time is uniform and around 30 s, regardless of the calibration satellite position [57]. Figure 12 shows the simulated total access time according to the relative position between L2-CalSat and CMB-Sat, considering again a FoV of $30^{\circ }$ and throughout 6 (a), 12 (b) and 24 h (c), but also for an expanded FoV of $120^{\circ }$ and throughout 12 h (d). As seen, for a FoV of $30^{\circ }$ and in the positions with maximum percentage of viewed sensors, about 30 min can be used for the calibration of pointing, polar angle, etc., in a calibration session of four precession periods (6 h), around one hour is available in a period of 12 h, and around two hours are available in a calibration session of 24 h. During the access time, in order to avoid the saturation of the detectors, the calibration signal power will be attenuated. In these calibration operations the Signal to Noise Ratios (SNR) provided by the sources on-board L2-CalSat have been estimated. Considering a Noise Equivalent Temperatures (NET) of the detectors in a range between 80 and 400 μK/$\sqrt{Hz}$ for the 40 to 400 GHz bandwidth, a fractional operational bandwidth of 30%, an integration time of 10 s, and a calibration signal power equal to half the saturation one, the calculated SNR values are between $4\times 10^{3}$ and $5.3\times 10^{4}$ (i.e., 36–47 dB). These values are similar to those estimated in a previous calibration satellite proposal for ground-based CMB experiments [11].

On the other hand, an expanded FoV of $120^{\circ }$ has also been considered to calculate the access time available for the characterization of the near- and far-side lobes of the beams. Figure 12d shows that in a calibration period of 12 h, a total access time of more than 5 h will be available for this task. Due to the large angular values of the far-side lobes, most of the sensors can be characterised with the calibration signal simultaneously, so the required time to calibrate all of them will be much lower than the available total access time. Taking into account that over a 3-year period of CMB mission, the total observation time of Jupiter (the most powerful celestial intensity calibrator) is of about one hour, and provide a NF range of about −40 to −70 dB from 40 to 400 GHz respectively, from Table 3 is easy to conclude that L2-CalSat can provide NF values better than −72 dB in all the frequency range, and in only 6 h (in fact, it is estimated that about 90 min of access time would be enough to characterise the FSL of all the sensors at the reported level of accuracy). Additionally, given the high SNR provided by the calibration sources of L2-CalSat, similar periods of time should be enough for both, intensity (beam pattern, non-linearity, bandwidth...) and polarization (beam pattern, polar angle...) in-flight calibration.

Finally, it is important to note that the electrical power system of the ancillary satellite allows to emit calibration signals only during a 40% of the calibration session period, because the rest of the time is necessary to recharge the battery module. However, this limitation does not represent an issue because, from the previous figures, it is estimated that for a calibration session of 12 h only about 2.5 h (1.5 for the beam characterization and 1 h for the polar angle, bandwidth, etc.) are required for calibration, which is about a 20% of the total calibration session time. As a consequence, during the periods of time in which the calibration signal is not reaching any sensor, the source will be turn-off and the battery will be recharged, in order to provide the required power to all the systems of L2-CalSat during the emission of the calibration signals.

## 6. Conclusions

A first design has been carried out for L2-CalSat, a calibration satellite for CMB space missions at the second Lagrange point of the Sun–Earth system. This design is in compliance with the requirements and objectives of a small-aperture CMB polarization mission, but can also be adapted to others, both in the microwave or in a different frequency range (optical, IR, etc.). The calibration satellite design has been developed at the CDF of the IDR/UPM in Madrid (Spain), following the CubeSat conceptual frame of implementation. The satellite and calibration sources have been described and a simple but efficient and secure calibration strategy has been proposed. It has been shown that L2-CalSat would reduce the polar angle error to the level of 1 arc-minute thanks to calibration sources providing SNR values between 36 and 47 dB. Also, it would allow the characterization of both, the intensity and polarization optical main beam and far-side lobes placed at $20^{\circ }$ or more from the center, thanks to NF levels lower than −72 dB provided in all the frequency range of interest. This level of accuracy can not be achieved by using celestial calibration sources as Tau-A or Jupiter due to the limited knowledge of the polarised emission and the reduced access time for intensity and polarization beam calibration, which affects mainly at lower frequencies. As a consequence, the use of a calibration system as the one proposed here would allow the exploitation of the high sensitivity provided by current and future CMB instrumentation and the achievement of ambitious scientific results.

## Figures and Tables

**Table 1:** Far Field Distance for different telescope apertures and frequency bands.

Aperture	40 GHz	60 GHz	90 GHz	150 GHz	220 GHz	350 GHz	650 GHz
0.3 m (LiteBIRD)	24 m	36 m	54 m	90 m	132 m	210 m	—
1.3 m (CORE)	451 m	676 m	1014 m	1690 m	2479 m	3943 m	6760 m
1.5 m (PICO)	600 m	900 m	1350 m	2250 m	3300 m	5250 m	9000 m

**Table 2:** Final payload characteristics.

Characteristics	Values	Units
Mass	1.3	[Kg]
Power Consumption (Average at Calibration)	50	[W]
Dimensions	20×10×10	[cm]
Temperature Range	0–50	[${}^{\circ }$C]

**Table 3:** Power arriving from Jupiter and L2-CalSat to a focal plane with 0.3 m aperture diameter. The maximum power of the calibration signal (second column), the thermal power from L2-CalSat (third column) and the thermal power from Jupiter (fourth column) are shown. Additionally, the power from CMB and the detectors saturation power are shown in the last two columns. All power values are given in pW. A Jupiter temperature of 160 K, fractional bandwidths of 0.25, Jupiter-Earth distance of 4.2 A.U. and distance between spacecrafts of 6600 m, have been considered in the calculations.

Freq. (GHz)	L2-CalSat (Cal.)	L2-CalSat (Th.)	Jupiter	CMB-Pow	Sat-Pow
40	40.8	3 × $10^{-4}$	2.2 × $10^{-3}$	0.16	0.5
100	5.1	4.7 × $10^{-3}$	34 × $10^{-3}$	0.21	0.8
200	5.1	37.5 × $10^{-3}$	0.27	0.12	0.9
400	5.1	0.3	2.17	15 × $10^{-3}$	1.2

**Table 4:** Final L2-CalSat characteristics.

Characteristics	Values	Units
Mass	7.193	[Kg]
Power Consumption (Maximum at Calibration)	62	[W]
Dimensions	30×20×10	[cm]
Temperature Range	0–50	[${}^{\circ }$C]

## References

[1] Tristram M., Banday A.J., Górski K.M., Keskitalo R., Lawrence C.R., Andersen K.J., Barreiro R.B., Borrill J., Eriksen H.K., Fernandez-Cobos R. (2021). Planck constraints on the tensor-to-scalar ratio. Astron. Astrophys..

[2] Akrami Y., Arroja F., Ashdown M., Aumont J., Baccigalupi C., Ballardini M., Banday A.J., Barreiro R.B., Bartolo N., Planck Collaboration (2020). Planck 2018 results. X. Constraints on inflation. Astron. Astrophys..

[3] Ade P.A.R., Ahmed Z., Aikin R.W., Alexander K.D., Barkats D., Benton S.J., Bischoff C.A., Bock J.J., BICEP2 Collaboration, Keck Array Collaboration (2018). Constraints on Primordial Gravitational Waves Using Planck, WMAP, and New BICEP2/Keck Observations through the 2015 Season. Phys. Rev. Lett..

[4] Kamionkowski M., Kovetz E.D. (2016). The Quest for B Modes from Inflationary Gravitational Waves. Annu. Rev. Astron. Astrophys..

[5] Debono I., Smoot G.F. (2016). General Relativity and Cosmology: Unsolved Questions and Future Directions. Universe.

[6] Padmanabhan T. (2015). One hundred years of General Relativity: Summary, Status and Prospects. arXiv.

[7] Iorio L. (2015). Editorial for the Special Issue 100 Years of Chronogeometrodynamics: The Status of the Einstein’s Theory of Gravitation in Its Centennial Year. Universe.

[8] Vishwakarma R.G. (2016). Einstein and Beyond: A Critical Perspective on General Relativity. Universe.

[9] Beltrán Jiménez J., Heisenberg L., Koivisto T.S. (2019). The Geometrical Trinity of Gravity. Universe.

[10] Aumont J., Macías-Pérez J.F., Ritacco A., Ponthieu N., Mangilli A. (2020). Absolute Calibration of the Polarisation Angle for Future CMB B-Mode Experiments from Current and Future Measurements of the Crab Nebula. Astron. Astrophys..

[11] Johnson B.R., Vourch C.J., Drysdale T.D., Kalman A., Fujikawa S., Keating B., Kaufman J. (2015). A CubeSat for Calibrating Ground-Based and Sub-Orbital Millimeter-Wave Polarimeters (CalSat). J. Astron. Instrum..

[12] Bonin G., Roth N., Armitage S., Newman J., Risi B., Zee R.E. CanX–4 and CanX–5 Precision Formation Flight: Mission Accomplished!. Proceedings of the 29th Annual AIAA/USU Conference on Small Satellites.

[13] Peters T.V., ao Branco J., Escorial D., Castellani L.T., Cropp A. (2014). Mission analysis for PROBA-3 nominal operations. Acta Astronaut..

[14] Bandyopadhyay S., Foust R., Subramanian G.P., Chung S.J., Hadaegh F.Y. (2016). Review of formation flying and constellation missions using nanosatellites. J. Spacecr. Rocket..

[15] Suzuki A., Ade P.A.R., Akiba Y., Alonso D., Arnold K., Aumont J., Baccigalupi C., Barron D., Basak S., Beckman S. (2018). The LiteBIRD Satellite Mission: Sub-Kelvin Instrument. J. Low Temp. Phys..

[16] Lee A., Ade P.A.R., Akiba Y., Alonso D., Arnold K., Aumont J., Austermann J., Baccigalupi C., Banday A.J., Banerji R. (2019). LiteBIRD: An all-sky cosmic microwave background probe of inflation. Bull. AAS.

[17] Hanany S., Alvarez M., Artis E., Ashton P., Aumont J., Aurlien R., Banerji R., Barreiro R.B., Bartlett J.G., Basak S. (2019). PICO: Probe of Inflation and Cosmic Origins. arXiv.

[18] Delabrouille J., de Bernardis P., Bouchet F., Achúcarro A., Ade P.A.R., Allison R., Arroja F., Artal E., Ashdown M., Baccigalupi C. (2018). Exploring cosmic origins with CORE: Survey requirements and mission design. J. Cosmol. Astropart. Phys..

[19] Delabrouille J., Abitbol M.H., Aghanim N., Ali-Haimoud Y., Alonso D., Alvarez M., Banday A.J., Bartlett J.G., Baselmans J., Basu K. (2019). Microwave Spectro-Polarimetry of Matter and Radiation across Space and Time. arXiv.

[20] Aghanim N., Akrami Y., Ashdown M., Aumont J., Baccigalupi C., Ballardini M., Banday A.J., Barreiro R.B., Bartolo N., Planck Collaboration (2020). Planck 2018 results. VI. Cosmological parameters. Astron. Astrophys..

[21] Kamionkowski M., Kosowsky A., Stebbins A. (1997). Statistics of cosmic microwave background polarization. Phys. Rev. D.

[22] Aghanim N., Akrami Y., Ashdown M., Aumont J., Baccigalupi C., Ballardini M., Banday A.J., Barreiro R.B., Bartolo N., Planck Collaboration (2020). Planck 2018 results. V. CMB power spectra and likelihoods. Astron. Astrophys..

[23] Ade P.A.R., Ahmed Z., Aikin R.W., Alexander K.D., Barkats D., Benton S.J., Bischoff C.A., Bock J.J., Bowens-Rubin R., Brevik J.A. (2016). Improved Constraints on Cosmology and Foregrounds from BICEP2 and Keck Array Cosmic Microwave Background Data with Inclusion of 95 GHz Band. Phys. Rev. Lett..

[24] Gerbino M., Lattanzi M. (2018). Status of Neutrino Properties and Future Prospects—Cosmological and Astrophysical Constraints. Front. Phys..

[25] Subramanian K. (2016). The origin, evolution and signatures of primordial magnetic fields. Rep. Prog. Phys..

[26] Minami Y., Ochi H., Ichiki K., Katayama N., Komatsu E., Matsumura T. (2019). Simultaneous determination of the cosmic birefringence and miscalibrated polarization angles from CMB experiments. Prog. Theor. Exp. Phys..

[27] Pogosian L., Shimon M., Mewes M., Keating B. (2019). Future CMB constraints on cosmic birefringence and implications for fundamental physics. Phys. Rev. D.

[28] Capparelli L.M., Caldwell R.R., Melchiorri A. (2020). Cosmic Birefringence Test of the Hubble Tension. Phys. Rev. D.

[29] Ade P.A.R., Aikin R.W., Barkats D., Benton S.J., Bischoff C.A., Bock J.J., Brevik J.A., Buder I., Bullock E., Dowell C.D. (2014). Detection of *B*-Mode Polarization at Degree Angular Scales by BICEP2. Phys. Rev. Lett..

[30] Ade P.A.R., Aghanim N., Ahmed Z., Aikin R.W., Alexander K.D., Arnaud M., Aumont J., Baccigalupi C., BICEP2/Keck Collaboration, Planck Collaboration (2015). Joint Analysis of BICEP2/Keck Array and Planck Data. Phys. Rev. Lett..

[31] Abazajian K., Addison G.E., Adshead P., Ahmed Z., Akerib D., Ali A., Allen S.W., Alonso D., Alvarez M., The CMB-S4 Collaboration (2020). CMB-S4: Forecasting Constraints on Primordial Gravitational Waves. arXiv.

[32] Hui H., Ade P., Ahmed Z., Aikin R., Alexander K., Barkats D., Benton S., Bischoff C., Bock J., Bowens-Rubin R. (2018). BICEP Array: A multi-frequency degree-scale CMB polarimeter. Millimeter, Submillimeter, and Far-Infrared Detectors and Instrumentation for Astronomy IX.

[33] Ade P., Aguirre J., Ahmed Z., Aiola S., Ali A., Alonso D., Alvarez M.A., Arnold K., Ashton P., Austermann J. (2019). The Simons Observatory: Science goals and forecasts. J. Cosmol. Astropart. Phys..

[34] Rubiño-Martín J.A., Rebolo R., Aguiar M., Génova-Santos R., Gómez-Reñasco F., Herreros J.M., Hoyland R.J., López-Caraballo C., Santos A.E.P., de la Rosa V.S., Stepp L.M., Gilmozzi R., Hall H.J. (2012). The QUIJOTE-CMB experiment: Studying the polarisation of the galactic and cosmological microwave emissions. Ground-Based and Airborne Telescopes IV.

[35] Nati F., Devlin M.J., Gerbino M., Johnson B.R., Keating B., Pagano L., Teply G. (2017). POLOCALC: A Novel Method to Measure the Absolute Polarization Orientation of the Cosmic Microwave Background. J. Astron. Instrum..

[36] Pérez I., Bosch-Lluis X., Camps A., Alvarez N., Hernandez J.F., Domènech E., Vernich C., De la Rosa S., Pantoja S. (2009). Calibration of Correlation Radiometers Using Pseudo-Random Noise Signals. Sensors.

[37] Hasebe T., Sekimoto Y., Dotani T., Mitsuda K., Shinozaki K., Yoshida S. (2019). Optimization of cryogenic architecture for LiteBIRD satellite using radiative cooling. J. Astron. Telesc. Instrum. Syst..

[38] Komatsu K., Matsumura T., Imada H., Ishino H., Katayama N., Sakurai Y. (2019). Demonstration of the broadband half-wave plate using the nine-layer sapphire for the cosmic microwave background polarization experiment. J. Astron. Telesc. Instrum. Syst..

[39] Bigot-Sazy M.-A., Charlassier R., Hamilton J.-C., Kaplan J., Zahariade G. (2013). Self-calibration: An efficient method to control systematic effects in bolometric interferometry. Astron. Astrophys..

[40] Adam R., Ade P.A.R., Aghanim N., Arnaud M., Ashdown M., Aumont J., Baccigalupi C., Banday A.J., Barreiro R.B., Planck Collaboration (2016). Planck 2015 results. VII. High Frequency Instrument data processing: Time-ordered information and beams. Astron. Astrophys..

[41] Bennett C.L., Larson D., Weiland J.L., Jarosik N., Hinshaw G., Odegard N., Smith K.M., Hill R.S., Gold B., Halpern M. (2013). Nine-year Wilkinson Microwave Anisotropy Probe (WMAP) Observations: Final Maps and Results. Astrophys. J. Suppl. Ser..

[42] Tauber J.A., Nielsen P.H., Martín-Polegre A., Crill B., Cuttaia F., Ganga K., Gudmundsson J., Jones W., Lawrence C., Meinhold P. (2019). Characterization of the in-flight properties of the Planck telescope. Astron. Astrophys..

[43] Takakura H., Sekímoto Y., Inatani J., Kashima S., Imada H., Hasebe T., Kaga T., Takeda Y., Okada N. (2019). Far-Sidelobe Antenna Pattern Measurement of LiteBIRD Low Frequency Telescope in 1/4 Scale. IEEE Trans. Terahertz Sci. Technol..

[44] Malphrus B.K., Freeman A., Staehle R., Klesh A.T., Walker R., Cappelletti C., Battistini S., Malphrus B.K. (2021). 4-Interplanetary CubeSat missions. Cubesat Handbook.

[45] GomSpace (2018). NanoSense Fine Sun Sensor (Datasheet, v2.2). https://gomspace.com/UserFiles/Subsystems/datasheet/gs-ds-nanosense-fss-22.pdf.

[46] Cubespace (2018). Cubestar Web Page. https://www.cubespace.co.za/products/adcs-components/cubestar/.

[47] Grelier T., Guidotti P.Y., Delpech M., Harr J., Thevenet J.B., Leyre X. Formation flying radio frequency instrument: First flight results from the PRISMA mission. Proceedings of the 2010 5th ESA Workshop on Satellite Navigation Technologies and European Workshop on GNSS Signals and Signal Processing (NAVITEC).

[48] Tethers-Unlimited (2020). RelNav Datasheet. https://tinyurl.com/yykneu7h.

[49] Colorado-University (2020). Colorado Web Page. https://www.colorado.edu/iriss/research-projects/atomic-project.

[50] Gomspace (2015). Gomspace Web Page. https://tinyurl.com/y6kdnbmh.

[51] Shufan Wu E.A. SULFRO: A Swarm of Nano-/Micro-Satellite at SE L2 for Space Ultra-Low Frequency Radio Observatory. Proceedings of the 28th Annual AIAA/USU Conference on Small Satellites.

[52] GomSpace (2018). NanoMind Z7000-On-board CPU and FPGA (Datasheet, v1.4). https://docplayer.net/40178505-Datasheet-on-board-cpu-and-fpga-for-space-applications.html.

[53] Virginia-Diodes (2020). VDI Web Page. https://vadiodes.com/en/frequency-multipliers.

[54] Vivatech (2018). Vivatech Web Page. http://vivatechmmw.com/en/.

[55] Farran-Technology (2020). Farran Web Page. https://www.farran.com/.

[56] O’Brient R. (2017). White Noise and Stability Study for the CMB Probe. TDM Option. http://hdl.handle.net/2014/48558.

[57] Bermejo-Ballesteros J., García-González S., Cubas J., Casas Reinares F.J., Martínez-González E. Development of a Calibration Satellite for a CMB Telescope Flying in Formation about L2 Libration Point. Proceedings of the 8th European Conference for Aeronautics and Space Sciences.

[58] Górski K.M., Hivon E., Banday A.J., Wandelt B.D., Hansen F.K., Reinecke M., Bartelmann M. (2005). HEALPix: A Framework for High-Resolution Discretization and Fast Analysis of Data Distributed on the Sphere. Astrophys. J..

[59] Lewis A., Challinor A. (2011). CAMB: Code for Anisotropies in the Microwave Background. http://ascl.net/1102.026.

